# Draft genome sequence of the halophilic bacterium *Chromohalobacter marismortui* DSM 6770^T^

**DOI:** 10.1128/mra.00873-26

**Published:** 2026-10-14

**Authors:** Terumipia Fujisawa-Phillips, T. B. K. Reddy, Vera Thiel, Markus Göker, Rekha Seshadri, Matthew Escobar

**Affiliations:** 1 Department of Biological Sciences, California State University San Marcos 14673, San Marcos, California, USA; 2 Department of Energy Joint Genome Institute, Lawrence Berkeley National Laboratory 1666, Berkeley, California, USA; 3 Leibniz Institute DSMZ, German Collection of Microorganisms and Cell Cultures, Braunschweig, Germany; University of Rhode Island, Kingston, Rhode Island, USA

**Keywords:** halophilic, ectoine, genome

## Abstract

*Chromohalobacter marismortui* DSM 6770^T^ is a moderately halophilic bacterium isolated from the Dead Sea in 1940. The *C. marismortui* DSM 6770^T^ draft genome is 3.55 Mbp, has 3,333 predicted genes, and includes a complete biosynthetic gene cluster for the production of the commercially significant osmoprotectant metabolites ectoine and 5-hydroxyectoine.

## ANNOUNCEMENT

Halophilic bacteria survive in hypersaline environments through various molecular mechanisms, including the accumulation of osmoprotectants like ectoine, a metabolite of significant commercial interest for its medical and biotechnological applications (1–3). *Chromohalobacter marismortui* DSM 6770^T^ (ATCC 17056^T^) is a rod-shaped, gram-negative, strictly aerobic, moderately halophilic bacterium isolated from the hypersaline Dead Sea in 1940 (4, 5). 16S rDNA analysis determined its taxonomic position in the family *Halomonadaceae* (6). Here, we present the draft genome sequence of *C. marismortui* DSM 6770^T^.

*C. marismortui* DSM 6770^T^ was grown aerobically in DSMZ medium 434 (7) at 37°C by the Leibniz Institute DSMZ. Genomic DNA was isolated using the MasterPure Gram-Positive DNA Purification Kit (Epicentre MGP04020) and is available through the DNA Bank Network (8). DNA was sequenced by the Joint Genome Institute (JGI) as part of the Genomic Encyclopedia of Type Strains Phase IV project. DNA (200 ng) was sheared to ~300 bp, and fragments underwent end-repair, A-tailing, and adapter ligation using a KAPA LTP Library Preparation Kit (KAPA Biosystems KK8230). The library was multiplexed and prepared for sequencing utilizing Illumina’s TruSeq Paired-end Cluster Kit (v4) and cBot instrument to generate a clustered flow cell. Sequencing was performed on an Illumina HiSeq 2500 using HiSeq TruSeq SBS sequencing kits (v4) with a 2 × 150 indexed run.

Sequencing generated 5,129,828 reads totaling 775 Mbp. Raw reads were filtered using BBDuk and BBMap (v39.82) (9) to trim adapters, remove reads ≤51 bp or containing “N” bases, and remove reads mapping to human, cat, dog, and mouse references, and common microbial contaminants at ≥93% identity. Genome assembly was performed using SPAdes (v3.10.1) with the following parameters: ––phred–offset 33––cov–cutoff auto –t 16 –m 115 ––careful –k 25,55,95 ––12 (10). A total of 200 bp was trimmed from contig ends, and contigs <1 Kbp or with read coverage <2× were discarded (BBTools [v39.82] reformat.sh: minlength). The final draft assembly contained 26 contigs in 23 scaffolds, totaling 3,553,220 bp. Genome annotation was performed using IMG Annotation Pipeline v4.15.2, as previously described (11). The Genomes OnLine Database system (12) was used to manage genome metadata, register BioProject and BioSample records at NCBI, and facilitate GenBank and Sequence Read Archive (SRA) submissions. Default parameters were used except where otherwise noted. Features of the *C. marismortui* DSM 6770^T^ draft genome are summarized in Table 1.

**TABLE 1:** Genome features of *C. marismortui* DSM 6770^T^

Feature	Value
Total scaffold sequence length (bp)	3,553,220
Number of scaffolds	23
Scaffold *N*_50_ (bp)	564,618
Average fold coverage (×)	214
CheckM2 genome completeness (%) (13)	99.99
Check M2 genome contamination (%) (13)	0.04
GC content (%)	61.71
Number of predicted genes	3,333
Number of protein-coding genes	3,264
Number of rRNA genes	4
Number of tRNA genes	56
JGI IMG/M taxon ID	2754412941
NCBI assembly accession number	GCA_004364315.1
NCBI BioProject accession number	PRJNA442672
NCBI SRA accession number	SRR7081649
NCBI BioSample number	SAMN08776302

**Fig 1:**
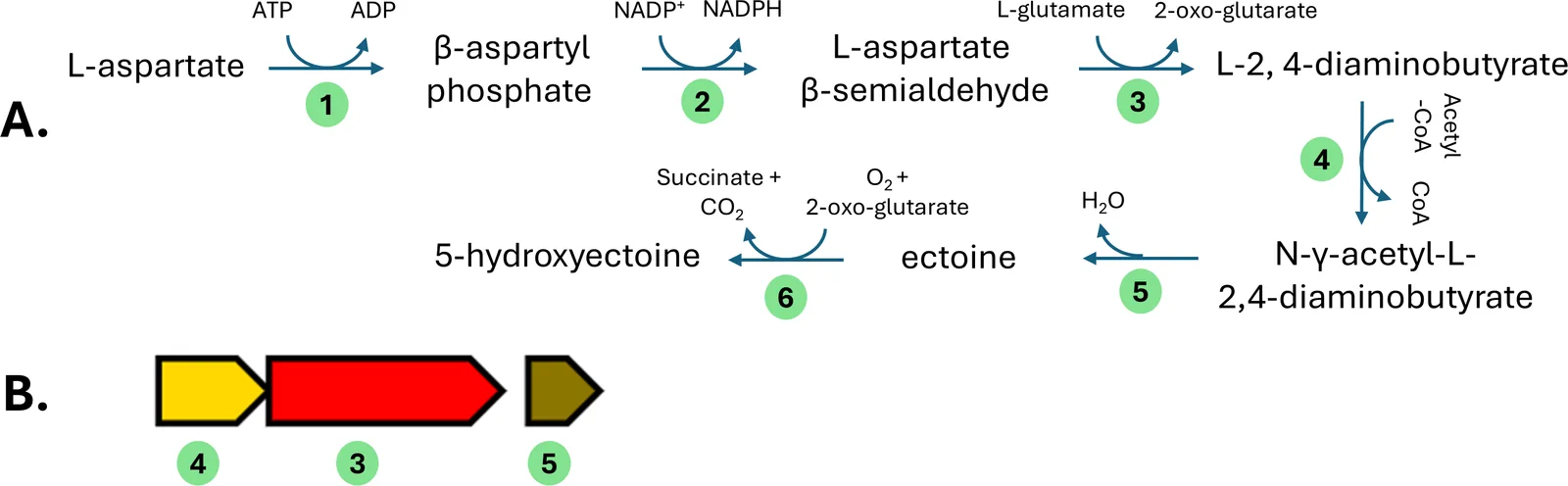
Ectoine/5-hydroxyectoine biosynthesis pathway in *C. marismortui* DSM 6770^T^. (**A**) Ectoine/5-hydroxyectoine synthesis pathway, with green circles indicating the presence of genes encoding biosynthesis pathway enzymes. 1: aspartate kinase (*Ask*), JGI Gene ID 2756151684; 2: aspartate-semialdehyde dehydrogenase (*Asd*), JGI Gene ID 2756153079; 3: diaminobutyrate-2-oxoglutarate transaminase (*ectB*), JGI Gene ID 2756151875; 4: L-2,4-diaminobutyric acid acetyltransferase (*ectA*), JGI Gene ID 2756151874; 5: L-ectoine synthase (*ectC*), JGI Gene ID 2756151876; and 6: ectoine hydroxylase (*ectD*), JGI Gene ID 2756153336. (**B**) Core ectoine biosynthetic gene cluster *ectA*/*B*/*C* within a single operon in the draft genome of *C. marismortui* DSM 6770^T^.

A previous study identified and partially sequenced a narrow host range plasmid, pCM1, from *C. marismortui* DSM 6770^T^ (14). This plasmid sequence, including two *repA* replication initiator genes and several viral-derived sequences (integrases, reverse transcriptase), is in genome scaffold Ga0215682_114 (17,027 bp). Production of the commercially valuable osmoprotectant ectoine has been verified in some *Chromohalobacter* species (15) and predicted based on genomic data in others (16). IMG/M and KEGG (17) analyses of the genome revealed a complete ectoine/5-hydroxyectoine biosynthesis pathway, with the core *ectA*/*B*/*C* genes located in a single operon (Fig. 1). Additional research will be necessary to confirm production of ectoine in *C. marismortui* DSM 6770^T^.

## Data Availability

This project has been deposited with the GenBank assembly accession number GCA_004364315.1. Additional publicly available genome resource links are included in Table 1.
